# Epidemic Bright's Disease

**Published:** 1909-03-27

**Authors:** 


### EPIDEMIC BRIGHT'S DISEASE.

Whether acute Bright's disease is due to the action of toxins or to the direct effects of micro-organisms is a point whose interest is at present chiefly academic. It is a very important advance, however, to have narrowed the question of the pathology of acute tubal nephritis down so far that one can say with fair certainty that it is caused by micro-organisms either directly or indirectly. A further advance has been to show that different organisms may produce similar lesions in the kidney with similar clinical symptoms and signs. It is true that quite a large number of fatal cases of acute tubal nephritis have been investigated bacteriologically with negative results. It is, however, equally true and far more important that acute tubal nephritis has been proved to have been caused in some cases by *streptococci*, in others by *pneumococci*, in others again by *bacilli coli communes*, and so on, the symptoms and urine changes in the pneumococcal cases being precisely similar to those of the streptococcal. It would seem not unlikely that yet other bacteria will be shown to have the power of producing that which we call acute tubal nephritis, for the varieties of micro-organisms are many, and the ways in which acute renal inflammation can manifest itself are comparatively few.

Be this as it may, if acute Bright's disease is ever due to micro-organisms or their products, one would expect that the malady might occur now and then in an epidemic form. That it sometimes does so is exemplified by the following cases:—

A man aged thirty-seven, a polisher by trade, came under observation in hospital on February 28, and gave the history of having been perfectly well until the previous August 22, when he developed intense headache and some soreness of the throat whilst at his work. Next day his face and feet were swelled up with œdema; the latter increased, and the patient took to his bed four days later in a condition of general anasarca that had persisted ever since. He was attended by a medical man, who examined him with every care. The sore throat was by that time entirely gone; there was no trace of any rash, nor had there been any; and there was neither then nor later any peeling or desquamation. The urine was repeatedly examined. It was the colour of beer, but never contained any quantity of blood; it was loaded with albumen, and abounded with various renal tube casts and epithelial cells. During the first fortnight of the illness the patient had severe epistaxis upon several occasions, diarrhœa with blood in the stools, and vomiting. He was treated in the usual way for acute nephritis, but the anasarca and albuminuria persisted, and by the following February, seven months after the beginning of the illness, the condition was that of chronic tubal nephritis with considerable œdema, ·6 parts of albumen per 1,000 of urine, renal cells and tube casts in abundance in the urinary deposit, and impairment of vision from albuminuric retinitis with small hæmorrhages.

In the present connection the chief point of interest in the case is the following history: the man had for five years been an employé in a workshop where the atmosphere was constantly moist and steamy. There were fourteen other men employed in the same place, working in groups. For eighteen months the patient had had the same two mates, all three working close to one another at the same table and often drinking from the same mug. One of these mates had complained of headache and sore throat about a week before the patient himself felt ill; and this man had come to the workshop the day after his sore throat began with his face and legs all puffy. He had been taken to a hospital, and had there died within a fortnight. The other mate had fallen ill at the same time as our patient, and, like him, he had first had headache, and then a day or two later œdematous swelling of the face and legs. He also had gone into a hospital, and was still there suffering from chronic tubal nephritis seven months later.

Here, therefore, were three persons, working together and living in daily contact, who were seized almost simultaneously with headache, sore throat, and anasarca, no other person in the same workshop having presented any similar symptoms. The sudden invasion, the distribution of the cases, the general phenomena and similarity of symptoms, all point to there having been a little epidemic of some infective or microbial disease prone to cause acute nephritis. A typical scarlet fever might be thought probable, but there is little or nothing to confirm such a suspicion. Scarlet fever was not known to be rife at the time. Diphtheria is another suggestion; the case was seen too late for bacterial investigations to be other than negative. Acute streptococcal tonsillitis seems quite as likely. It is impossible to say now what microbe was at work, but the cases seem to confirm the view that acute nephritis is indirectly or directly microbic in origin.

